# Chlorantraniliprole resistance associated with diamondback moth (Lepidoptera: Plutellidae) outbreaks in Arizona *Brassica* crops

**DOI:** 10.1093/jee/toae212

**Published:** 2024-09-24

**Authors:** Wilfrid Calvin, John C Palumbo

**Affiliations:** Department of Entomology, Yuma Agricultural Center, University of Arizona, Yuma, AZ 85364, USA; Department of Entomology, Yuma Agricultural Center, University of Arizona, Yuma, AZ 85364, USA

**Keywords:** diamide, insecticide resistance, resistance management, *Brassica* transplants

## Abstract

The diamondback moth, *Plutella xylostella* (Linnaeus), is one of the most important insect pests of *Brassica* crops worldwide. In October 2016, outbreaks of an invasive *P. xylostella* population and unexpected control failures occurred on broccoli and cauliflower crops throughout all vegetable-growing regions in Arizona. Nineteen populations of *Plutella xylostella* were collected from 2016 to 2021 from various commercial cauliflower fields in Yuma and Scottsdale, Arizona, and from experimental broccoli plots at the University of Arizona, Yuma Agricultural Center (UAYAC), Yuma, Arizona. Populations collected from the commercial cauliflower fields had been transplanted with seedlings produced in a local Yuma nursery in 2016 and Salinas, CA in 2017 to 2018, whereas experimental broccoli plots were direct seeded. These populations were evaluated for their susceptibility to chlorantraniliprole, spinetoram, emamectin benzoate, and cyantraniliprole. In this study, field rate laboratory bioassays, serial dilution laboratory bioassays, and field efficacy spray experiments were performed. The field rate laboratory bioassay results showed that spinetoram, emamectin benzoate, and cyantraniliprole remained effective at controlling *P. xylostella,* but chlorantraniliprole did not control *P. xylostella* at the field rate. Additionally, serial dilution bioassays confirmed significant levels of cyantraniliprole and chlorantraniliprole resistance in the *P. xylostella* populations collected from transplanted cauliflower fields. However, the results of the multiyear/growing-seasons study monitoring the susceptibility of *P. xylostella* populations collected from direct-seeded broccoli and field efficacy trials conducted at the UAYAC indicated that the resistance to diamide insecticides was neither uniform nor persistent following the 2016 outbreak. Nevertheless, the risk for *P. xylostella* resistance in Arizona vegetable-growing regions exists, particularly in *Brassica* transplants. Therefore, we recommend that Arizona *Brassica* growers remain vigilant and practice rigorous insecticide resistance management to offset the development of resistance.

## Introduction

The diamondback moth, *Plutella xylostella*, is one of the most important insect pests of *Brassica* crops worldwide and annually causes billions of dollars in economic losses associated with yield reductions and increased control costs (Zalucki et al. 2012, Furlong et al. 2013). *Plutella xylostella* larvae feed on leaves and marketable portions of all *Brassica* crops and can cause significant yield losses if not adequately managed (Shelton 2001). Under outbreak conditions, controlling larvae and adults can be very difficult, often requiring multiple insecticide applications. In Arizona, the second leading producer of broccoli and cauliflower in the United States, these crops were grown on 19,980 acres with a market value of approximately $241 million in 2023 (USDA-NASS, 2023). *Plutella xylostella* is typically considered a minor pest of these *Brassica* cultivars in Arizona and rarely reaches economic levels. Currently, growers can easily control the pest with 1 to 2 well-timed insecticide sprays. However, during the 2016 to 2017 growing season, unexpected outbreaks of *P. xylostella* and widespread control failures occurred on *Brassica* crops throughout all Arizona vegetable-growing regions (Palumbo 2021).

*Plutella xylostella* has a well-documented history of resistance to most insecticide chemistries (Tabashnik et al. 1987, 1990, Shelton et al. 2000, Zhao et al. 2006, Santos et al. 2011, Gong et al. 2014, Agboyi et al. 2016, Roy et al. 2023). In recent years, resistance to chlorantraniliprole, a diamide insecticide, has been reported in *P. xylostella* populations from several parts of the world, including China (Wang and Wu 2012, Wang et al. 2013, Gong et al. 2014), Brazil (Filho et al. 2023), and India (Yeole et al. 2018). Additionally, a decline in susceptibility to chlorantraniliprole in *P. xylostella* populations collected from 2016 to 2019 across Georgia and Florida was also reported (Riley et al. 2020, Dunn et al. 2021). During the 2016 to 2017 growing season, failure to control *P. xylostella* populations in Yuma and Scottsdale, Arizona was thought to be associated with chlorantraniliprole resistance (Palumbo 2021).

Insecticide rotation is strongly recommended for *P. xylostella* insecticide resistance management (Zhao et al. 2010, Riley and Sparks 2017). However, because diamide insecticides are very effective against lepidopteran insect pests in comparison to other alternative insecticides and fit well in most crop-growing systems, these insecticides have been extensively used, causing extreme selection pressure in lepidopteran populations, including *P. xylostella* (Richardson et al. 2020). Several mechanisms of chlorantraniliprole resistance in *P. xylostella* populations have been reported. Some researchers have reported mutation in the target site as the cause of chlorantraniliprole resistance in some *P. xylostella* populations (Troczka et al. 2012, Richardson et al. 2020, Dunn et al. 2021), whereas, in other *P. xylostella* populations, metabolic resistance has been found to be the mechanism of resistance (Mallot et al. 2019). The differences in resistance mechanisms among *P. xylostella* populations may render the management of the pest even more challenging and can influence the choice of resistance management strategies (Tabashnik et al. 1997).

The objective of this study was to characterize the susceptibility of *P. xylostella* populations to chlorantraniliprole collected from heavily infested cauliflower and broccoli fields in Arizona in an attempt to understand the causes of the 2016 outbreak. The study also consisted of the evaluation of *P. xylostella* populations collected from both heavily infested cauliflower and broccoli fields and from University of Arizona Yuma Agricultural Center (UAYAC) experimental fields for their susceptibility to and gather baseline information on four commonly used insecticides.

## Materials and Methods

### Insects and Colony Culture

Nineteen populations of *P. xylostella* were collected from 2016 to 2021 from various commercial cauliflower fields in Yuma and Scottsdale, Arizona, and from experimental broccoli plots at the UAYAC, Yuma, Arizona. Populations from commercial fields in 2016 to 2018 were collected where growers had complained of unusually heavy *P. xylostella* infestations and/or difficulty in controlling larvae. Larvae in 2017 and 2018 were collected from fields planted with transplants grown in Salinas, CA, whereas collections in 2016 were from fields planted from transplants grown in a local nursery. At each site, approximately 100 *P. xylostella* larvae and pupae were collected and transported to the UAYAC laboratory and placed in culture for bioassays. A susceptible *P. xylostella* laboratory strain from Dupont Crop Protection (Newark, DE) was also used in the 2016 bioassays for comparison. For each colony, adults that emerged from the field populations were provided a 10% honey solution in cages with freshly collected cabbage leaves for oviposition and replaced daily. Emerged larvae were reared on a modified artificial diet (Southland Products Inc., Little Village, AR) containing 10% pre-prepared desiccated cabbage flour powder. Third instar larvae of the F_1_ or F_2_ generation were used for all bioassays. All populations were maintained at 25 ± 2 °C, 50% to 60% RH, and a photoperiod of 14:10 (L:D) h during rearing and all laboratory bioassays.

### Insecticides

Formulated insecticides used for bioassays and field trials included spinetoram (Radiant SC, 120 g ai/L; Dow Agrisciences, Indianapolis, IN), emamectin benzoate (Proclaim, 5% SG; Syngenta Crop Protection, Greensboro, NC), chlorantraniliprole (Coragen SC, 200 g ai/L) DuPont Crop Protection, Newark, DE), cyantraniliprole (Exirel SE, 100 g ai/L; DuPont Crop Protection, Newark, DE) and a pre-mixture of chlorantraniliprole + lambda-cyhalothrin (Besiege SC, 100 g ai/L + 50 g ai/L; Syngenta Crop Protection, Greensboro, NC).

### Field Rate Laboratory Bioassays

In the fall of 2016, initial tests were conducted in sprayed-leaf and leaf-dip bioassays to determine the relative mortality of field-collected *P. xylostella* larvae exposed to standard field rates of commonly used insecticides. Second and third instar larvae collected from two heavily infested commercial cauliflower fields (Yuma Valley Co. 9, and Co. 14) in November 2016 were transported to the laboratory and held on nontreated cauliflower heads for 1 day before bioassay.

For the leaf dip bioassay, each insecticide solution was prepared using distilled water containing 0.1% Dyne-Amic (Helena Agri-enterprises, LLC, Collierville, TN), which is equivalent to the recommended field rate applied at 468 l/ha (50 GPA). Cauliflower, *Brassica oleracea* L., leaf disks measuring 9.0 cm in diameter were cut and dipped in each insecticide solution for 30 s, allowed to dry for 1 h, and placed in petri dishes. Control disks were treated with a solution 0.1% Dyne-Amic and distilled water. Ten 3rd instar *P. xylostella* larvae were placed in each Petri dish with four replications per insecticide treatment (*n* = 40). Petri dishes were maintained in a climate-controlled room at 25 ± 2 °C, 50% to 60% RH, and a photoperiod of 14:10 (L:D) h and arranged in a randomized complete block design (RCBD). *Plutella xylostella* mortality was assessed 96 h after being exposed to insecticide treatments. The larvae were considered dead when they were unable to move when being probed with a soft paintbrush.

In the sprayed-leaf bioassay, insecticides were applied to foliage at recommended field rates commonly used by local growers. Cauliflower leaf disks measuring 9.0 cm in diameter were cut and placed on paper towels outside in a fallow field. A foliar spray of each compound was applied to the adaxial surface of 4 leaf disks using a CO_2_-pressurized backpack sprayer calibrated to deliver 234 ha/L (25 GPA), similar to the treatment of plants in small plots efficacy trials (Palumbo 2022). All insecticide solutions included an adjuvant, Dyne-Amic, @ 0.125% (v/v). The insecticide-treated leaf disks were allowed to air dry for 1 h in the field and then transported to the laboratory, where ten 3rd instar *P. xylostella* larvae were placed on each treated leaf disk in Petri dishes with 4 replications per insecticide treatment (*n* = 40). The mortality of *P. xylostella* larvae was assessed using the same criteria described in the above leaf-dip bioassays.

### Serial Dilution Laboratory Bioassays

Insecticide susceptibility of *P. xylostella* larvae populations collected from the commercial cauliflower fields and experimental broccoli plots (UAYAC) was assessed using an IRAC-approved leaf dip bioassay (IRAC 2010). Prior to bioassay, dosages were determined in preliminary assays using 0.01, 0.1, 1.0, 10, and 100 mg ai/L solutions to determine the proper serial dilutions for each compound. For the formal bioassays, 5 to 6 concentrations of each formulated compound that caused mortality ranging from 5% to 95% were selected. Leaf disks measuring 9 cm in diameter were cut from cabbage leaves, dipped individually in the serial dilutions of each insecticide treatment for 30 s with gentle agitation, and placed on paper towel and allowed to dry for 1 h. Control leaf discs were dipped individually in a solution comprised of distilled water and 0.1% Dyne-Amic. The dried leaves were placed on filter paper within glass Petri dishes. Ten 3rd instar *P. xylostella* larvae were placed on each disk in each Petri dish with 4 replications per insecticide treatment (*n* = 40). *Plutella xylostella* mortality was evaluated 72 h after inoculation for spinetoram and emamectin benzoate, and 96 h for the diamides as per the IRAC methodology (IRAC 2010). As previously described, *P. xylostella* larvae were considered dead if they could not move when being prodded using a soft paintbrush. Bioassays with control mortality exceeding 3% were excluded.

### Field Efficacy Spray Experiments

In each growing season (fall and spring plantings) from 2016 to 2021, 2 small plot field experiments were conducted to measure the relative insecticide efficacy against *P. xylostella* larvae under local field conditions. In each experiment, the broccoli variety *Emerald Crown* was direct-seeded into double-row beds on 106.68-m (42-in.) centers in September for the fall experiments and December for the spring experiments at the UAYAC. The plots were 2 beds wide and 10.67 m (35 ft) long and bordered by 2 nontreated beds. Four replications of each treatment were arranged in an RCBD. The formulations and rates used for the tested insecticides were as described in Table 1. Insecticide applications were initiated at the first detection of *P. xylostella* larvae in the plots. Three foliar sprays were applied on 10 to 14 d intervals with a CO_2 -_pressurized boom sprayer calibrated to deliver a broadcast application through 2 TXVS-18 ConeJet nozzles per bed at 344.7 kPa (50 Psi) and 210.5 L/ha (22.5 gal/ac). An adjuvant, Dyne-Amic, was added to each treatment at a rate of 0.125% (v/v). For efficacy assessment, after 6 to 7 days following each insecticide application, 10 plants were randomly selected from each replicate and destructively sampled (plants were removed from the field) for the presence of larvae. The nontreated checks in each of these fall and spring experiments served as the source of *P. xylostella* larvae used in the serial dilution bioassays.

**Table 1.** Efficacy of Radiant (spinetoram), Proclaim (emamectin benzoate), Coragen (chlorantraniliprole), Besiege (chlorantraniliprole + lambda-cyhalothrin), and Exirel (cyantraniliprole) using standard field-rates against 2 field collected populations of *P. xylostella* larvae in laboratory, leaf dip and sprayed-leaf bioassays, Fall 2016

		Mean (± SEM) % larval mortality, 96 h after exposure			
		Leaf dip bioassay (468 l/ha)		Sprayed-leaf bioassay (234 l/ha)	
Insecticide	Field rate/ac equivalent	Yuma Valley, Co. 14^a^	Yuma Valley, Co. 9^a^	Yuma Valley, Co. 14	Yuma Valley, Co. 9
Radiant SC	5 fl oz	100 (0.0) a	93.8 (6.2) a	72.5 (6.3) a	81.9 (6.4) a
Proclaim 5SG	4.8 oz	100 (0.0) a	71.9 (11.8) a	84.2 (7.1) a	83.5 (5.7) a
Exirel 0.83SE	13.5 fl oz	97.3 (2.2) a	84.4 (9.4) a	77.5 (4.8) a	81.7 (10.6) a
Coragen 1.67SC	5 fl oz	60.5 (6.3) b	14.6 (6.9) b	15.5 (3.2) b	12.5 (5.5) b
Besiege SC	9 fl oz	58.3 (2.5) b	15.6 (6.0) b	15.5 (3.2) b	10.1 (2.3) b
Non-treated check	—	2.5 (2.5) c	2.5 (2.5) b	2.5 (2.5) b	5.0 (3.8) b

Means within columns for each bioassay followed by a common letter are not significantly different (Tukey’s HSD, α = 0.05).

^a^Yuma Valley, County 14th street population, and Yuma Valley, County 9th street population.

### Statistical Analysis

Results of the serial dilution bioassays for each insecticide were analyzed with probit analysis using the PoloSuite program (Russell et al. 1977, LeOra Software 2016). Data are presented as LC_50_ values (µg ai/mL) with 95% fiducial limits (FLs) for each insecticide. Differences in susceptibility between populations for an insecticide were determined based on non-overlapping 95% FLs. The resistance ratio (RR) was calculated by dividing the LC_50_ value of a field-collected population by the LC_50_ value of the YAYAC-Fall 2016 population for spinoteram, emamectin benzoate, and chlorantraniliprole and the LC_50_ value of the YAYAC-Fall 2017 population for cyantraniliprole. The UAYAC populations were considered as the susceptible field population and were used in place of the susceptible population from Dupont Crop Protection in our resistance ratio calculations and susceptibility determinations. Because the *P. xylostella* laboratory strain was extremely susceptible, a comparison using this strain was unrealistic. For the field rate bioassays, the mortality response data were analyzed using an analysis of variance (ANOVA) (PROC GLIMMIX; SAS Institute 2009). The mortality response data for each *P. xylostella* strain was subjected to an arcsine transformation prior to the analysis to account for heterogeneity (Zar 1999). Because of the heterogeneity of mean variances in the field efficacy spray experiments, data for total *P. xylostella* larvae per 10 plants (averaged across the 3 applications) were transformed using a log_10_ (*x* + 1) function before being analyzed using an ANOVA. Treatment means were separated using Turkey’s HSD test (*P *≤ 0.05). Means from non-transformed data are presented.

## Results

### Field Rate Laboratory Bioassays

In the leaf dip bioassay, significant differences in percent larval mortality among the evaluated insecticides were detected for both Yuma Valley, Co. 14 (*F* = 47.83; df = 5, 23; *P* < 0.0001) and Yuma Valley Co. 9 (*F* = 31.21; df = 5, 23; *P* < 0.0001) *P. xylostella* populations (Table 1). All insecticides evaluated in the Yuma Valley Co. 14 population resulted in significantly greater larval mortality than the nontreated check. Radiant SC, Proclaim 5SG, and Exirel 0.83SE provided comparable larval mortalities and exhibited significantly greater larval mortalities than Coragen 1.67SC and Besiege SC (Table 1). For the Yuma Valley Co. 9 population, only Radiant SC, Proclaim 5SG, and Exirel 0.83SE exhibited *P. xylostella* mortalities significantly greater than that of both Coragen 1.67SC and Besiege SC, which were not significantly different from the nontreated check (Table 1). The sprayed-leaf bioassay results were consistent with the leaf dip bioassay except that the larval mortality caused by Coragen and Besiege did not differ from the nontreated check for either the Yuma Valley Co. 14 population (*F* = 38.17; df = 5, 23; *P* < 0.0001) or the Yuma Valley, Co. 9 populations (*F* = 16.08; df = 5, 23; *P* < 0.0001) (Table 1).

### Serial Dilution Laboratory Bioassays

*Emamectin benzoate*: In the bioassays evaluating emamectin benzoate, the *P. xylostella* populations collected from Scottsdale in the fall of 2016, Yuma Valley, Co. 14 in the fall of 2016, Mohawk Valley in the fall of 2018, and Yuma Valley, Co. 12 in the fall of 2018 exhibited LC_50_ values and resistance ratios significantly greater than that of the UAYAC population based on non-overlapping LC_50_ Fiducial Limits (FLs; Table 2). However, *P. xylostella* populations collected from Mohawk Valley, Yuma Valley, Co. 9, Yuma Valley Co. 11, and Yuma Valley Co. 13 in the fall of 2016 had emamectin benzoate susceptibility levels comparable to that of the UAYAC population. We also monitored the susceptibility of the *P. xylostella* UAYAC populations collected from direct-seeded broccoli to emamectin benzoate from Fall 2016 through Spring 2021. Our data demonstrated that the susceptibility level within the UAYAC populations mostly remained significantly unchanged except for the population collected in the Spring of 2019, which exhibited an LC_50_ value and resistance ratio significantly greater than that of the UAYAC population collected in the fall of 2016 (Table 3).

**Table 2.** Relative toxicity of emamectin benzoate (Proclaim) against Arizona field populations of *P. xylostella*

Population	Collection date	*n* ^a^	Generation	Slope (± SEM)	LC_50_ (95% FL) [mg ai/mL]	*x* ^2^	Resistance ratio (RR)^b^
UAYAC	Fall 2016	200	F_1_	1.33 (0.17)	0.26 (0.09-0.75)	1.60	1.00
Scottsdale	Fall 2016	200	F_2_	1.64 (0.25)	1.23 (0.88-1.71)	0.20	4.73*
Mohawk Valley	Fall 2016	200	F_2_	1.32 (0.17)	0.87 (0.39-1.95)	1.10	3.35
Yuma Valley, Co. 9	Fall 2016	200	F_1_	1.53 (0.21)	0.71 (0.21-2.42)	2.10	2.73
Yuma Valley, Co. 11	Fall 2016	200	F_1_	1.21 (0.16)	1.35 (0.51-3.60)	1.20	5.20
Yuma Valley, Co. 13	Fall 2016	200	F_2_	1.34 (0.19)	0.83 (0.33-2.16)	1.10	3.20
Yuma Valley, Co. 14	Fall 2016	200	F_1_	1.39 (0.22)	5.84 (4.85-7.11)	0.60	22.46*
Mohawk Valley	Fall 2018	240	F_1_	1.47 (0.20)	2.55 (2.10-3.10)	0.10	9.81*
Yuma Valley, Co.12	Fall 2018	240	F_1_	2.35 (0.38)	3.93 (3.46-4.46)	0.50	15.12*

^a^Total number of larvae assayed.

^b^Resistance ratios were calculated by dividing the LC_50_ value of the test population by the LC_50_ value of the UAYAC-Fall 2016 population.

*Significant resistance ratios based on non-overlapping 95% FLs.

**Table 3.** Toxicity of emamectin benzoate (Proclaim) against UAYAC field populations of *P. xylostella* across growing seasons and years relative to UAYAC-Fall 2016 population of *P. xylostella*

Population	*n* ^a^	Generation	Slope (± SEM)	LC_50_ (95% FL) [mg ai/mL]	*x* ^2^	Resistance ratio (RR)^b^
UAYAC-Fall 2016	200	F_1_	1.33 (0.17)	0.26 (0.09-0.75)	1.60	1.00
UAYAC-Spring 2017	240	F_2_	1.30 (0.16)	1.55 (0.71-2.20)	2.90	5.96
UAYAC-Fall 2017	240	F_2_	1.48 (0.22)	0.11 (0.09-0.13)	0.10	0.42
UAYAC-Fall 2018	240	F_1_	1.24 (0.16)	0.47 (0.33-0.67)	0.20	1.80
UAYAC-Spring 2019	240	F_1_	1.11 (0.15)	3.12 (1.22-9.90)	2.60	12.00*
UAYAC-Fall 2019	240	F_2_	1.39 (0.19)	0.15 (0.09-0.26)	0.50	0.58
UAYAC-Spring 2020	240	F_1_	1.26 (0.17)	0.29 (0.12-0.62)	0.40	1.12
UAYAC-Fall 2020	240	F_2_	1.40 (0.19)	0.24 (0.11-3.9)	4.70	0.92
UAYAC-Spring 2021	240	F_1_	1.92 (0.28)	0.48 (0.34-0.66)	0.30	1.85

^a^Total number of larvae assayed.

^b^Resistance ratios were calculated by dividing the LC_50_ value of the test population by the LC_50_ value of the UAYAC-Fall 2016 population.

^*^Significant resistance ratios based on non-overlapping 95% FLs.

*Spinetoram*: Most *P. xylostella* populations evaluated were susceptible to spinetoram relative to the UAYAC populations collected in the fall of 2016, except for the Mohawk Valley population collected in the fall of 2018, which were slightly less susceptible to spinetoram based on non-overlapping LC_50_ Fiducial Limits (FLs; Table 4). Consistently, monitoring the susceptibility of the UAYAC populations of *P. xylostella* to spinetoram from fall 2016 through Spring 2021 demonstrated that relative to the UAYAC population collected in the fall of 2016, the UAYAC populations remain susceptible to spinetoram for most of the growing seasons except for the population collected in the spring of 2019 (Table 5). Interestingly, *P. xylostella* UAYAC populations collected in the falls of 2017 and 2020 and spring of 2021 exhibited a 4.17- to 6.25-fold increase in susceptibility to spinetoram (Table 5).

**Table 4.** Relative toxicity of spinetoram (Radiant) against Arizona field populations of *P. xylostella*

Population	Collection date	*n* ^a^	Generation	Slope (± SEM)	LC_50_ (95% FL) [mg ai/mL]	*x* ^2^	Resistance ratio (RR)^b^
UAYAC	Fall 2016	240	F_1_	1.37 (0.17)	4.51 (2.08-9.84)	2.80	1.00
Scottsdale	Fall 2016	240	F_2_	1.32 (0.18)	12.15 (2.88-54.6)	6.50	2.68
Mohawk Valley	Fall 2016	240	F_2_	1.39 (0.18)	9.1 (2.82-28.11)	5.10	2.02
Yuma Valley, Co. 9	Fall 2016	240	F_1_	1.11 (0.16)	16.51 (7.06-46.92)	2.80	3.66
Yuma Valley, Co. 11	Fall 2016	240	F_1_	0.80 (0.11)	15.99 (3.39-240.26)	6.10	3.55
Yuma Valley, Co. 13	Fall 2016	240	F_2_	0.91 (0.12)	2.5 (1.07-6.21)	1.90	0.55
Yuma Valley, Co. 14	Fall 2016	240	F_1_	1.07 (0.13)	7.11 (3.90-16.90)	2.20	1.58
Mohawk Valley	Fall 2018	240	F_1_	1.67 (0.25)	27.74 (14.70-56.50)	2.30	6.15*
Yuma Valley, Co.12	Fall 2018	240	F_1_	1.66 (0.24)	25.17 (7.90-112.40)	11.10	5.58

^a^Total number of larvae assayed.

^b^Resistance ratios were calculated by dividing the LC_50_ value of the test population by the LC_50_ value of the UAYAC-Fall 2016 population.

^*^Significant resistance ratios based on non-overlapping 95% FLs.

**Table 5.** Relative toxicity of spinetoram (Radiant) against UAYAC field populations of *P. xylostella* across growing seasons and years relative to UAYAC-Fall 2016 population of *P. xylostella*

Populations	*n* ^a^	Generation	Slope (± SEM)	LC_50_ (95% FL) [mg ai/mL]	*x* ^2^	Resistance ratio (RR)^b^
UAYAC-Fall 2016	240	F_1_	1.37 (0.17)	4.51 (2.08-9.84)	2.80	1.00
UAYAC-Spring 2017	240	F_2_	1.30 (0.17)	11.20 (2.4-24.2)	3.30	2.48
UAYAC-Fall 2017	240	F_2_	2.08 (0.33)	0.70 (0.47-1.02)	0.60	0.16^*^
UAYAC-Fall 2018	240	F_1_	1.44 (0.19)	5.17 (3.3-7.9)	1.00	1.11
UAYAC-Spring 2019	240	F_1_	0.89 (0.15)	66.30 (34.42-172.2)	0.94	14.7^*^
UAYAC-Fall 2019	240	F_1_	1.48 (0.19)	1.12 (0.40-2.70)	3.80	0.25
UAYAC-Spring 2020	240	F_1_	1.29 (0.15)	1.84 (0.40-11.50)	6.10	0.41
UAYAC-Fall 2020	240	F_2_	1.23 (0.14)	0.73 (0.50-1.20)	1.10	0.16^*¤^
UAYAC-Spring 2021	240	F_1_	1.56 (0.21)	1.07 (0.90-1.30)	0.20	0.24^*¤^

^a^Total number of larvae assayed

^b^Resistance ratios were calculated by dividing the LC_50_ value of the test population by the LC_50_ value of the UAYAC-Fall 2016 population.

^*^Significant resistance ratios based on non-overlapping 95% FLs.

^¤^Populations with a resistance ratio significantly lower than the reference population.

*Chlorantraniliprole*: All evaluated *P. xylostella* populations exhibited chlorantraniliprole LC_50_ values and resistance ratios significantly greater than that of the UAYAC population collected in the fall of 2016, with resistance ratios ranging from 16.62 to 60.84 (Table 6). We have monitored chlorantraniliprole susceptibility within *P. xylostella* UAYAC populations from fall 2016 through spring 2021; in the spring of 2017, we observed a 25.44-fold decrease in susceptibility in UAYAC populations, which did not carry over in the next 2 growing seasons (Table 7). However, in the fall of 2018 and spring of 2019, we observed another 18.23- and 11.79-fold decrease in susceptibility in the *P. xylostella* UAYAC population, which again did not persist through the following growing seasons until Spring of 2021 (Table 7).

**Table 6.** Relative toxicity of chlorantraniliprole (Coragen) against Arizona field populations of *P. xylostella*

Population	Collection date	*n* ^a^	Generation	Slope (± SEM)	LC_50_ (95% FL) [mg ai/mL]	*x* ^2^	Resistance ratio (RR)^b^
UAYAC	Fall 2016	240	F_2_	0.71 (0.08)	9.70 (2.60-36.30)	3.60	1.00
Scottsdale	Fall 2016	240	F_2_	2.56 (0.46)	590.10 (579.00-603.60)	0.30	60.84*
Mohawk Valley	Fall 2016	240	F_3_	2.21 (0.34)	392.50 (362.80-424.10)	0.30	40.46*
Yuma Valley, Co. 9	Fall 2016	240	F_1_	2.51 (0.47)	588.00 (579.90-596.30)	1.20	60.62*
Yuma Valley, Co. 13	Fall 2016	240	F_2_	2.09 (0.33)	411.40 (250.50-667.50)	2.70	42.41*
Yuma Valley, Co. 14	Fall 2016	240	F_1_	1.09 (0.15)	190.20 (75.80-523.70)	9.80	19.61*
Mohawk Valley	Fall 2018	240	F_1_	1.55 (0.20)	161.20 (129.40-198.20)	0.40	16.62*
Yuma Valley, Co.12	Fall 2018	240	F_1_	2.25 (0.38)	485.90 (370.00-640.90)	1.10	50.09*

^a^Total number of larvae assayed.

^b^Resistance ratios were calculated by dividing the LC_50_ value of the test population by the LC_50_ value of the UAYAC-Fall 2016 population.

^*^Significant resistance ratios based on non-overlapping 95% FLs.

**Table 7.** Relative toxicity of chlorantraniliprole (Coragen) against UAYAC field populations of *P. xylostella* across growing seasons and years relative to UAYAC-Fall 2016 population of *P. xylostella*

Population	*n* ^a^	Generation	Slope (± SEM)	LC_50_ (95% FL) [mg ai/mL]	*x* ^2^	Resistance ratio (RR)^b^
UAYAC-Fall 2016	240	F_2_	0.71 (0.08)	9.70 (2.60-36.30)	3.60	1.00
UAYAC-Spring 2017	240	F_2_	1.26 (0.17)	246.80 (46.70-903.70)	6.90	25.44*
UAYAC-Fall 2017	240	F_2_	0.98 (0.12)	1.33 (0.49-3.20)	4.80	0.14
UAYAC-Spring 2018	288	F_1_	0.75 (0.10)	2.90 (0.17-9.50)	4.70	0.30
UAYAC-Fall 2018	240	F_1_	1.32 (0.17)	176.80 (90.80-330.90)	2.60	18.23*
UAYAC-Spring 2019	240	F_1_	1.51 (0.18)	114.40 (39.80-268.20)	5.30	11.79*
UAYAC-Fall 2019	240	F_1_	0.81 (0.13)	1.80 (0.03-7.30)	4.90	0.19
UAYAC-Spring 2020	240	F_1_	0.82 (0.10)	7.60 (1.90-19.90)	2.90	0.78
UAYAC-Fall 2020	240	F_2_	0.70 (0.09)	6.10 (1.90-13.70)	1.60	0.63
UAYAC-Spring 2021	240	F_1_	1.21 (0.17)	5.90 (1.40-17.40)	1.70	0.61

^a^Total number of larvae assayed.

^b^Resistance ratios were calculated by dividing the LC_50_ value of the test population by the LC_50_ value of the UAYAC-Fall 2016 population.

^*^Significant resistance ratios based on non-overlapping 95% FLs.

*Cyantraniliprole*: Due to the lack of *P. xylostella* larvae for some populations, only 3 populations were evaluated for cyantraniliprole. Like chlorantraniliprole, both populations from commercial fields evaluated exhibited LC_50_ values and resistance ratios (20.81- and 15.71-fold) significantly higher than that of the UAYAC population collected in the fall of 2016 based on non-overlapping LC_50_ Fiducial Limits (FLs) (Table 8). Our susceptibility monitoring bioassays demonstrated a moderate decrease in susceptibility to cyantraniliprole in UAYAC *P. xylostella* populations collected in the fall of 2018 and spring of 2019. This decrease in susceptibility disappeared for the next 3 growing seasons, with another slight reduction in susceptibility observed in spring 2021 (Table 9).

**Table 8.** Relative toxicity of cyantraniliprole (Exirel) against Arizona field populations of *P. xylostella*

Population	Collection date	*n* ^a^	Generation	Slope (± SEM)	LC_50_ (95% FL) [mg ai/mL]	*x* ^2^	Resistance ratio (RR)^b^
UAYAC	Fall 2017	240	F_2_	1.71 (0.32)	0.97 (0.50-1.40)	1.10	1.00
Mohawk Valley	Fall 2018	240	F_1_	1.94 (0.22)	20.19 (15.80-25.90)	0.90	20.81*
Yuma Valley, Co.12	Fall 2018	240	F_1_	1.85 (0.22)	15.24 (6.00-37.10)	7.70	15.71*

^a^Total number of larvae assayed.

^b^Resistance ratios were calculated by dividing the LC_50_ value of the test population by the LC_50_ value of the UAYAC-Fall 2017 population.

^*^Significant resistance ratios based on non-overlapping 95% FLs.

**Table 9.** Relative toxicity of cyantraniliprole (Exirel) against UAYAC field populations of *P. xylostella* across growing seasons and years relative to UAYAC-Fall 2017 population of *P. xylostella*

Population	*n* ^a^	Generation	Slope (± SEM)	LC_50_ (95% FL) [mg ai/mL]	*x* ^2^	Resistance ratio (RR)^b^
UAYAC-Fall 2017	240	F_2_	1.71 (0.32)	0.97 (0.50-1.40)	1.10	1.00
UAYAC-Spring 2018	288	F_1_	1.53 (0.26)	1.29 (0.80-1.80)	0.60	1.33
UAYAC-Fall 2018	240	F_1_	1.75 (0.21)	7.64 (2.86-17.10)	6.90	7.88*
UAYAC-Spring 2019	240	F_1_	2.48 (0.30)	11.54 (8.20-16.20)	8.20	11.9*
UAYAC-Fall 2019	240	F_1_	1.34 (0.21)	1.98 (0.30-4.50)	5.20	2.04
UAYAC-Spring 2020	240	F_1_	1.31 (0.25)	0.81 (0.15-1.60)	2.10	0.84
UAYAC-Fall 2020	240	F_2_	1.34 (0.26)	1.35 (0.60-2.20)	0.50	1.40
UAYAC-Spring 2021	240	F_1_	2.87 (0.45)	6.12 (4.76-7.77)	1.20	6.31*

^a^Total number of larvae assayed.

^b^Resistance ratios were calculated by dividing the LC_50_ value of the test population by the LC_50_ value of the UAYAC-Fall 2017 population.

^*^Significant resistance ratios based on non-overlapping 95% FLs.

### Field Efficacy Spray Experiments

**Fig. 1.**
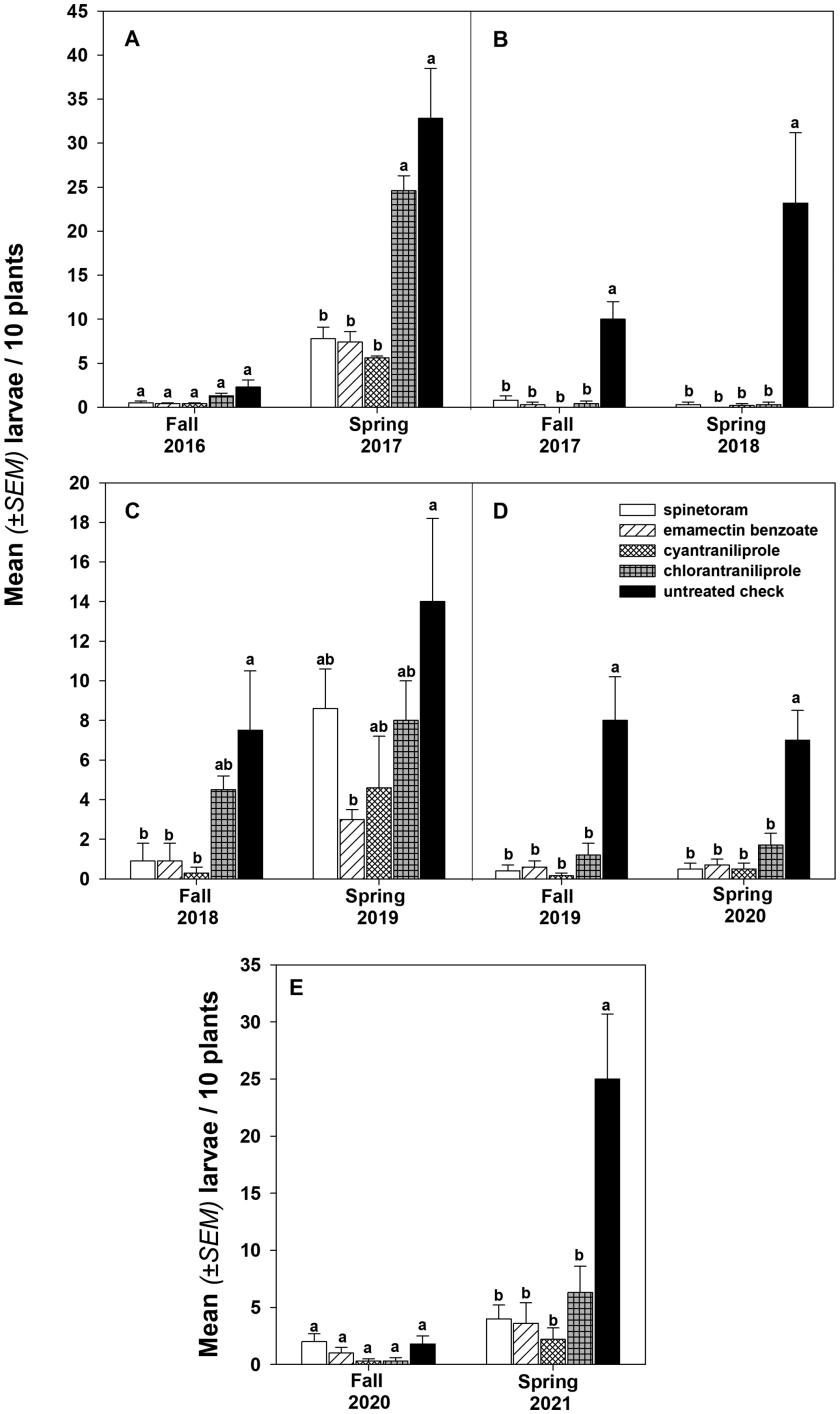
Efficacy of spinetoram, emamectin benzoate, cyantraniliprole, and chlorantraniliprole against *P. xylostella* in small-plot, broccoli field trials conducted at UA Yuma Agricultural Center, 2016-2021. A: Fall 2016 and Spring 2017 growing seasons; B: Fall 2017 and Spring 2018 growing seasons; C: Fall 2018 and Spring 2019 growing seasons; D: Fall 2019 and Spring 2020 growing seasons; E: Fall 2020 and Spring 2021 growing seasons. Means followed by the same letter in each trial are not significantly different (Tukey’s HSD, α = 0.05).

In the fall of 2016, there were no significant differences in *P. xylostella* larvae mortality among treatments and the nontreated check in the direct-seeded broccoli plots. However, in Spring 2017, while spinetoram, emamectin benzoate, and cyantraniliprole provided significant control of *P. xylostella* larvae relative to the nontreated check, larval incidence in plots treated with chlorantraniliprole did not differ from that of the nontreated check (Fig. 1A). During the following seasons (Fall 2017 and Spring 2018), all insecticides evaluated provided measurable control of *P. xylostella* relative to the nontreated and were comparable to each other (Fig. 1B). Similar to the spring of 2017, in the fall of 2018 spinetoram, emamectin benzoate, and cyantraniliprole provided measurable control of the pest, but chlorantraniliprole had level of larval incidence that did not differ to that of the nontreated check (Fig. 1C). In the subsequent growing season, spring of 2019, only emamectin benzoate provided satisfactory control of the *P. xylostella* larvae relative to the nontreated check (Fig. 1C). In the fall of 2019 and spring of 2020, all evaluated insecticides (spinetoram, emamectin benzoate, cyantraniliprole, and chlorantraniliprole) provided effective control of *P. xylostella* relative to the nontreated check and exhibited comparable level of control (Fig. 1D). In the fall of 2020 growing season there was very low pressure of *P. xylostella* which prevented us from observing differences among the insecticide treated plots and the nontreated check. In the spring of 2021, all evaluated insecticides provided measurable control of *P. xylostella* larvae (Fig. 1E). Results from the field efficacy experiment data are very consistent with what we have observed in our serial dilution bioassays for the UAYAC populations.

## Discussion

In Arizona, *P. xylostella* is a minor pest that occasionally reaches damaging levels that requires insecticide intervention in fall and spring *Brassica* crops. However, in October 2016, outbreaks of an invasive *P. xylostella* population and unexpected control failures occurred on broccoli and cauliflower crops throughout all vegetable-growing regions in Arizona. Control failures continued throughout the remainder of the spring 2017 growing season. To address the nature of this incident, we conducted laboratory and field studies using four commonly applied insecticides in Arizona crops, including emamectin benzoate, spinetoram, cyantraniliprole, and chlorantraniliprole. We initially conducted field rate bioassays on populations from heavily infested cauliflower fields to determine whether *P. xylostella* showed any signs of reduced susceptibility to field-equivalent rates of these compounds. These assays strongly suggested that chlorantraniliprole was not providing control at the field rate. Further, serial dilution bioassays consistently confirmed diamide resistance associated with the *P. xylostella* outbreaks experienced in broccoli and cauliflower fields during the fall of 2016-spring 2017 growing season.

Overall, our studies indicated that emamectin benzoate and spinetoram remained very effective at controlling *P. xylostella* populations collected across the vegetable-growing regions in Arizona. This was consistent with reports from growers and pest control advisors (PCAs) who noted good larval control following applications of these compounds. However, all *P. xylostella* populations evaluated exhibited significant resistance levels to the diamides, cyantraniliprole and chlorantraniliprole. In the same period (2016 to 2019), a similar trend was reported in *P. xylostella* populations collected across Georgia and Florida (Riley et al. 2020, Dunn et al. 2021), indicating that the occurrence of resistance in *P. xylostella* populations in Arizona was not an isolated incidence.

The development of these resistant *P. xylostella* populations are unlikely to have originated from vegetable-growing regions in Arizona because the pest does not survive Arizona summers (May–August) when *Brassica* crops and weeds are not available as plant hosts. The host range of *P. xylostella* is well known to be limited to only plants within the family Cruciferae (Talekar and Shelton 1993, Sarfraz et al. 2006) and *Brassica* weeds do not grow during the summer in Yuma and Scottsdale due to high temperatures and dry conditions (Parker 1972). This is further supported with extensive year-round pheromone trapping data from 2017-2021 that clearly demonstrated the lack of adult moths captured during the summer months, only to reappear in the early fall (Palumbo 2021). This strongly suggests that new *P. xylostella* populations infesting *Brassica* crops each fall in Arizona either migrate via winds associated with monsoon or tropical storms from neighboring *Brassica* growing regions in Sonora and Baja California, Mexico that annually occur during August and September, or arrive into the region on infested cauliflower transplants from California (Palumbo 2021). In Arizona, greater than 95% of the cauliflower crop is transplanted with seedlings produced in the Yuma area or shipped in from coastal California nurseries.

The *P. xylostella* outbreaks in 2016 initially occurred only on transplanted cauliflower crops and it was quickly determined that the source of the invasive populations originated from infested seedlings grown in one local Yuma nursery. The transplant nursery could not control the *P. xylostella* in their growing facilities, and transplants were delivered to the affected fields heavily infested with *P. xylostella* larvae. Anecdotal reports indicated that insecticides with chlorantraniliprole as an active ingredient had been extensively used in this facility to control *P. xylostella* larvae. Additionally, PCAs and growers indicated that within a few weeks of transplanting seedlings supplied by this local facility, they had difficulty controlling the *P. xylostella* infestations, particularly with diamide insecticides. Nearby direct-seeded broccoli fields and cauliflower fields grown using transplants originating from southern California did not report issues with *P. xylostella* (Bill Fox and Brad Brchan, personal communications).

After the first local transplanted fields began to be harvested in November 2016, several growers reported that seriously infested fields suffered significant yield reductions and incurred extremely high control costs (Palumbo 2017). By late December 2016, *P. xylostella* populations began to spread from the infested transplanted fields to nearby direct-seeded *Brassica* (i.e., broccoli and kale) crops throughout the region, causing further significant losses. By February 2017, areawide reports of infested broccoli and cauliflower fields were common.

The *P. xylostella* infestations experienced by Arizona growers in 2016 to 2017 were not anticipated, and it was suspected that a resistant population had become established in the local nursery during the previous summer (Palumbo 2017). It is speculated that the initial source of the diamide-resistant *P. xylostella* population was brought into the local nursery on plants shipped from coastal CA prior to transplanting in Yuma, spread throughout the facility, and exposed to repeated applications of chlorantraniliprole and other insecticide chemistries. A similar case was observed in New York, where *P. xylostella* outbreaks occurred due to infested cabbage transplants shipped from Florida, Georgia, and Maryland greenhouses. Some of these *P. xylostella* populations were highly resistant to commonly used insecticides such as permethrin and methomyl, which led to control issues and yield losses (Shelton et al. 1996).

Results of our multiyear/growing-seasons study monitoring the susceptibility and field efficacy of *P. xylostella* populations collected at the UAYAC to the 4 insecticides since the outbreak until Spring 2021 further indicates that the resistance to diamide insecticides was consistent and not generated in the field, but from locally grown transplants. Because the *P. xylostella* populations do not thrive in between *Brassica* growing seasons (May–August), it would not be possible for resistance genes to be transferred to new *P. xylostella* populations migrating into Arizona growing regions the following fall season (Talekar and Shelton 1993, Zhao et al. 2006).

Surveys of growers and PCAs serving Arizona and southern California further support these findings. In the subsequent growing seasons following the 2016 outbreaks, they were able to control the pest with diamide insecticides using an average of 1 and 1.6 applications for broccoli/cauliflower and transplanted cabbage, respectively, compared to multiple sprays applied in 2016-2017 (Palumbo 2017, 2021). Despite the climatic conditions in the growing regions in Arizona that are very conducive for insect activity and the intensity of agriculture in the regions, the evolution of resistance is very rare in pest populations. This situation is particularly due to crop diversification and/or rotation, such as vegetable and leafy greens production during fall, winter, and spring growing seasons, and melons, cotton, alfalfa, Sudan grass, etc., in the summer months. Thus, *P. xylostella* populations do not survive the summer due to a lack of appropriate food source availability (i.e., *Brassica* crops and weeds), which plays a significant role in *P. xylostella* resistance management (Palumbo 2021). In essence, *P. xylostella* populations found each fall in Arizona crops are not the same populations that occurred on *Brassica* crops the previous growing season. In contrast, Zhao et al. (2006) reported that resistance to spinosad in California and Georgia resulted from extensive applications made throughout the year to a common *P. xylostella* population found on continuous sequential plantings of collards in adjacent fields or farms. Not surprising, the first cases of resistance to a diamide insecticide occurred in Southeast Asia and believed to be caused by a lack of crop rotation and over-reliance on the mode of action (MOA; Richardson et al. 2020).

In conclusion, we found that resistance to diamide insecticides suddenly occurred in several *P. xylostella* populations in crop-growing regions of Arizona due to the introduction of cauliflower transplants infested with chlorantraniliprole-resistant larvae. Although the resistance did not persist, it is a warning that it is necessary to implement an effective insecticide resistance management (IRM) program for the diamide insecticides to remain effective not only against *P. xylostella* but all target pests affecting crops grown in Arizona and beyond. It is crucial to maintain the long-term efficacy of chlorantraniliprole. This insecticide is a suitable choice for producers because, in addition to being effective, it has little negative impact on insect pests’ natural enemies (Richardson et al., 2020).

Our observations throughout this study reaffirm the importance of implementing IRM tactics. *Plutella xylostella* populations that establish at the beginning of each fall growing season in Arizona crops do not inherit the genetic mutations that occurred in *P. xylostella* population from the previous spring season. However, growers must be aware that populations migrating on coastal California transplants or storms from northern Mexico may possibly be resistant to chlorantraniliprole. This has become increasingly important as serial dilution bioassays of *P. xylostella* larvae collected from Salinas, Santa Maria, and Camarillo, CA from 2018-2021 showed chlorantraniliprole resistance associated with reported field control failures following applications of Coragen (chlorantraniliprole) to *Brassica* crops (JCP, unpublished data). This likely explains the chlorantraniliprole resistance detected in the Mohawk Valley and Yuma Valley, Co. 12 populations in the Fall 2018 (Table 6) collected from fields with cauliflower transplants originating from Salinas.

Thus, it is highly recommended that Arizona growers proactively implement IRM tactics to effectively manage *P. xylostella* and mitigate insecticide resistance in all transplanted and direct-seeded *Brassica* crops. These tactics include proper communication with the transplant providers regarding pest issues and MOA used to control them, thorough inspection of transplants before planting, preventive control of the pests with an effective systemic insecticide prior to transplanting, frequent field scouting, proper crop sanitation, conservation of natural enemies, and rotation of insecticide MOA (Talekar and Shelton 1993, Palumbo 2023).
